# Pemphigus

**Published:** 1882-06

**Authors:** Leon Sandberg

**Affiliations:** Russia


PEMPHIGUS. By LEON SANDBERG, of Russia. Translated from the German by F. H. GEUTSCH, M.D., Chicago.

Pemphigus belongs, undoubtedly, to that class of skin diseases about whose nature we are thus far but little instructed. It manifests itself, on the one hand, in a great variety of forms and manifold complications, and on the other, by its rarity of occurrence.

Whether this disease was known to the ancients or not, we find mentioned in the works of Hypocrates a febrile disease, under the name of febris pemphigoides, in the course of which pustules were developed in the bucal cavity. There prevailed in all the ancient works, until the end of the last century, so much confusion upon this obscure disease, that we can accept them as of but little value. For the first lucid description of this disease we have to thank Lepois and Willan. The credit for its new classification belongs to Wichmann. Instead of the five classes of Savage, viz.: Pemphigus major, Castrensis, Helveticus, Indicus and Brasiliensis, he classified them as simply acute and chronic.

According to the French dermatologists, we have the classification, Pompholix congenital, solitaire, confluent, simultaneous, successive, acute chronic, pyretique, apyretique and herpes phlyctenoide. Hardy, in opposition, gives two principal forms, acute and chronic, but gives their different manners of invasion. Cazenaave sets up a new form, which differs from the ordinary chronic pemphigus in this, that separate pustules follow rapidly one upon the other, and with equal rapidity burst. In this form the pustules spread rapidly from the first point of invasion over the whole body. The pustules are small and not well filled, so that the coverings lie in folds. The red color of the contents of the pustule comes from the infected cuticle at its base. After the falling of the scab, a moist spot remains, which has no tendency to heal. These spots give off a fatty smell, and are very painful. This form received from Cazenaave the name of pemphigus foliaceus.

Whilst most dermatologists regard acute pemphigus as a distinct disease, Hebra teaches that every true pemphigus runs a chronic course. This question can only be settled by careful observation at the bedside. From the best information we can gather in regard to the so-called acute pemphigus, we see that it is not peculiar to children alone, but is found also, though rarely, in adults.

The statement of Bärensprung, that in the acute pemphigus, pustules form also upon the mucous membranes similar to those upon the skin, seems to be borne out by the facts in most cases, as in the case described by Coston, to which we will refer again later, in which this eruption was very well pronounced. This case can be cited as an instance of the occurrence of acute pemphigus, as the eruption was preceded by a distinct prodromic stage of two days' duration, in the same manner as in the acute exanthemata. Later, Heraud observed three cases which ran an acute course, attended with high fever. Ulmer kept one of his cases, after it had run its acute course, under his eye for a considerable length of time, to see whether there would be any further manifestations of the disease, but failed to note any.

It is otherwise in regard to the contagious character of pemphigus.

The well-marked epidemics of pemphigus which have at times presented themselves stand in contradictory relation with the barren results of inoculation with the contents of the pemphigus pustule, so that we can hesitate to accept them alone as sufficient evidence of the contagious nature of the disease, as it is by many regarded. Also, in the case of Scharlan, in which the contents of the pustule should have shown contagious properties; viz.: that a number of persons who had been handling newly born children afflicted with the disease, also subsequently acquired an eruption, can still not be regarded as proof of its contagious nature. Notwithstanding the convincing evidence of the results of his inoculation by the pustulous matter, it still leaves a doubt whether it was not syphilis he was dealing with. Even though we know the course and various complications of this disease, we are still in the dark as to its etiology, and the light which a knowledge of the part taken by the internal organs might throw upon it.

Thus is the nature of this dangerous disease still obscure, in spite of the energy and labors of our most eminent dermatologists. In order to invite investigation into the nature of pemphigus, I send an account of a case of pemphigus foliaceus whose course seems particularly important.

A patient forty-two years old, badly nourished, suffered in his childhood from a scrofulous enlargement of the cervical glands, which at one time formed an abscess and required the use of the knife. In later years he suffered frequently from inflammation of the throat. The patient belonged to a sickly family, but in which there had never been any skin disease. His father died of lung disease, and his mother of apoplexy. Out of seven brothers and sisters six died; one brother living and well. The patient was obliged to give up his trade, that of a shoemaker, because he could not endure the sedentary life, and has been for six years employed in a bone mill. No appearance of venereal disease. Excused from military duty as unfit. Was twice married, and had with the first wife six children, four of whom died in childhood; two are living and well. From the second marriage there are two children, both living and well.

In July, 1877, there appeared upon his limbs, without previous warning, a pustulous eruption, which in twenty-four hours spread over his entire body, when the pustules enlarged and became confluent; afterward ruptured, leaving moist and painful spots. At the time of his admission to the Julius hospital, October 1, 1877, his whole body was covered with the charactereristic jagged shreds of skin of pemphigus. Over the whole surface of the skin were scattered flabby and sunken pustules, varying in size from a pea to a bean, filled with a milky fluid. On various parts of the body, particularly on the face and back, were crusts of eczema, under which lay the corium. Pustules also appeared in the external ear. The conjunctiva and mucous membrane were free from pustules, but lachrymation was profuse. Nothing abnormal found about the thoracic viscera or liver, or spleen. The assimilative powers were bad, with loss of appetite and diarrhœa. Under treatment with permanent baths of caustic potash, and arsenic internally, the patient improved. His weight increased from 50 to 64 kilograms. April 7, 1878, he was discharged convalescent.

During his stay in the hospital no albumen was found in the urine, but large quantities of phosphates.

Soon after leaving the hospital the pemphigus returned, with the same rapidity and intensity, covering the whole body, so that on June 27 he returned to the hospital. He was subjected to the permanent baths, and during the night Hebra's salve applied, and later penciled with pyrogallic acid. Internal administration of arsenic, morphia and hydrate of chloral to relieve pain. After an improvement in his general health, and of the skin disease, the patient again left the hospital, February 25, 1879.

Five weeks later the disease made its appearance once more, the patient being then engaged in the very arduous labor of breaking stone. At this third appearance in the hospital the disease presented the same phase as in the first instance. The palpebral conjunctiva much inflamed, with epiphora ; the outer ear excoriated and covered with pustules. Upon the forehead appeared circumscribed red spots denuded of epidermis; on the periphery of the spots were shreds of skin, as upon other parts of the body. The only remedy which proved efficacious in relieving the external symptoms was the permanent bath, twenty-four hours after using which flabby pustules appeared between those npon the epidermis. The nutrition sank considerably meanwhile. Diarrhœa alternated with constipation. The therapeutics same as for previous periods, with the exception that the pyrogallic acid seemed this time to have little effect. No fever. Heavy precipitate in the urine, but no albumen.

August 3, 1880. Patient felt somewhat better. Breast and back sparsely covered with pustules; the pustules already dried and burst, and the epidermis hanging in jagged shreds from the excoriated parts. The patient was kept all day in permanent bath, and at six P. M. was placed in bed, after the excoriated parts had been covered with oiled cloths. Appetite and sleep normal, but patient complains of continuous itching in the extremities.

August 17. An addition of corrosive sublimate to the bath had to be given up after two days, on account of the appearance of salivation. The urine, which was examined every six days, was found free of albumen, rich in urates, and of brownish yellow color.

In the latter part of October the appetite failed, and vomiting began.

November 3, the patient experienced several chills, and pneumonia of the left lung.

November 7. Another severe chill. The temperature varied between 39 and 40.3 C. until the third stage of the pneumonia, November 20.

November 16, a new outbreak of pustules was noticed. On the lower part of the breast and the entire surface of the abdomen the epidermis detached itself in patches as large as the hand, formed by the confluence of mucous pustules, and thus exposing the corium, which, together with the remaining contents of the pustules, formed yellow, bad smelling scabs. The same condition of the back rendered the recumbent position very painful.

November 20. With the decline of the fever, the skin of the breast and back began to assume its normal appearance. No more pustules appeared on the back, but upon the ocular conjunctiva of the left eye pustules as large as a pea developed themselves, which, on opening or closing the eye, acted like a foreign body. Patient was once more placed in the permanent bath. His appetite improved, and weight increased from 42.5 to 61 kilograms.

About the end of January, 1881, a new eruption made its appearance, attended by fever; the temperature at times rising as high as 39.7 C.

At this time the patient presented a typical case of pemphigus foliaceus. Upon the whole body were numerous pustules, some filled with pus and others with serum, and covered with scales of epidermis. Upon the nates were cracks and chaps which were easily caused to bleed, and which made the dorsal decubitus very painful. In contrast with the rest of the body, the palms of the hands and the soles of the feet presented a normal appearance, and remained free from eruption during the whole course of the disease. In March the patient began to complain of pains in the mouth and of loss of appetite, and rapidly lost flesh. On inspection of the bucal cavity, we found the tongue red and denuded of the epithelial coat, and a crucial-shaped coating on its surface, and the margins also coated. Upon the point of the tongue were three pustules, the size of a pea, and filled with a transparent liquid, each of which pustules was surrounded by a number of minute ones of the same character. The gums and jaws were normal in appearance. The skin began to wear a better appearance. The breast and upper extremities were almost free of eruption. The back and lower extremities also showed no pustules, but scales of epidermis similar to those resulting from ruptured blisters, and which were arranged in circular form upon the body. Upon the hairy scalp and upon both ears were attached with dried pus the same sort of scales, forming with the hair a white crust, and giving the appearance of flour having been strown over the scabs or crusts.

In April the treatment by permanent baths had to be discontinued, on account of the aversion of the patient to them. After two months, in consequence of lying with dressed limbs, a slight contraction of the knee joints appeared. Forcible extension was not applied, on account of the severe pain produced, but passive motion was freely employed.

In October the condition of the patient was worse. Severe pains in the head, and tearing and drawing pains in the right hip joint. The inguinal glands became swollen, but not tender on pressure. Over the lower portion of the abdomen, as well as upon the right thigh, many new pustules appeared. Those upon the thigh soon became confluent. The epidermis became detached from the entire surface of the thigh and the upper third of the leg. The contents of the pustules, at first serous, assumed the character of pus. After the appearance of the pains in the hip, the temperature rose, the breathing became difficult, and, owing to the debility of the patient, expectoration impossible. In the beginning of November there appeared, attended with severe burning pains, new pustules upon the left lower extremity, which ran the same course as those upon the right thigh. The knees on both sides were contracted at right angles. Shortly before death, the patient began to complain of pains in the left breast. Percussion showed a region of dullness of two fingers breadth in the posterior lower portion of the left lung. Auscultation revealed pleuritic friction sounds. The heart impulse grew weaker, and on November 10 death ensued from paralysis of the heart.

Post-mortem examination one day after death revealed the following, viz.: Muscles dark and dry. Adipose membrane much reduced. In the left heart, clots of dark blood in large quantities; same in the right; no ante-mortem clots; valves normal. Brown coloration of the muscles of the heart, and reduced in quantity; left endocardium of light color. Anterior part of right lung normal, but posterior part of upper half of same partially solidified; patches of ecchymosis upon the pleura. Hyperæmia of the trachæa and larger bronchial tubes. There was less infiltration of the left lung than of the right one. Considerable deposits of fibrin upon the pleura; in the upper lobe a deposit of tubercles. Bronchial mucous membrane on this side less hyperæmic than upon the other. Stomach distended, and upon the lesser curvature a simple ulcer, with a swollen base, seemed to be in process of healing; upon the opposite side were cicatrices of old ulcers. The subserous coat of the small intestines much injected; the mesentery light-colored and swollen, with thickening of the mesocolon. The mesenteric glands between colon and ilium were enlarged to the size of a pigeon's egg, and cheesy. Slight erosion of the lower part of the large intestine; over the valvula Bauhini ulcerative changes. Spleen large and white, and few malpigian bodies; liver enlarged, with fatty infiltration of its borders. Right kidney normal in size; left one enlarged with light-colored cortical substance.

Microscopical examination of the brain, spinal cord and sympathetic revealed nothing abnormal.

To recapitulate: We have here to deal with a delicate man of forty-two years, who was scrofulous in his childhood, and who lived under bad influences. His sickness began in July, 1877, and caused his death four years afterward.

With the most patients, we find with this disease a tendency to exhaustion and general disturbance of the digestive apparatus. The intensity and extent of the skin affections are variable, and at times the patients experience intervals of almost entire freedom from them. While in most cases the eruption is not attended with fever, yet in some cases it is preceded by high fever.

Ten days before death, this patient had marked enlargement of the inguinal glands, attended with severe pain in the right hip joint, and also high fever of a remittent type, which until death remained as high as 39 to 40.3 C., the remissions amounting, some days, to 3° C. In this case, every sign of kidney disease, which in the etiology of pemphigus with the older authors played so important a *rôle*, was wanting; also those signs of nervous disturbance to which, in the last decade, the origin of pemphigus has so often been attributed, were wanting. Still, we find in this case some moments, even though they appear to be merely coincident or accidental, which we may perhaps point to as the origin of the disease. There is no doubt that the constitution of the individual, in this disease, as in many others, has much to do with the origin of the disease. Most victims of this disease are broken-down, debilitated individuals, who live under bad influences, and who are frequently exposed to wet and cold.—*Münchener Medicinisehe Wochenschrift.*

---

To make a differential diagnosis between the inter-costal neuralgias and pleural or pulmonary disease, observe two facts: The neuralgias are associated not only with pain, but also with tenderness on pressure; the pulmonary processes by pain alone. The second fact is that neuralgias are unattended by fever; the reverse prevails in the opposite conditions.—*E. T. Bruen, in Pocket-Book of Phys. Diagnosis.*

